# Ecological Niche Modeling for the Prediction of the Geographic Distribution of Cutaneous Leishmaniasis in Tunisia

**DOI:** 10.4269/ajtmh.15-0345

**Published:** 2016-04-06

**Authors:** Bilel Chalghaf, Sadok Chlif, Benjamin Mayala, Wissem Ghawar, Jihène Bettaieb, Myriam Harrabi, Goze Bertin Benie, Edwin Michael, Afif Ben Salah

**Affiliations:** The Centre for Research and Applications in Remote Sensing, Department of Applied Geomatics, Sherbrooke University, Quebec, Canada; Eck Institute for Global Health, University of Notre Dame, Notre Dame, Indiana; Laboratory of Medical Epidemiology, Pasteur Institute of Tunis, Tunis, Tunisia

## Abstract

Cutaneous leishmaniasis is a very complex disease involving multiple factors that limit its emergence and spatial distribution. Prediction of cutaneous leishmaniasis epidemics in Tunisia remains difficult because most of the epidemiological tools used so far are descriptive in nature and mainly focus on a time dimension. The purpose of this work is to predict the potential geographic distribution of *Phlebotomus papatasi* and zoonotic cutaneous leishmaniasis caused by *Leishmania major* in Tunisia using Grinnellian ecological niche modeling. We attempted to assess the importance of environmental factors influencing the potential distribution of *P. papatasi* and cutaneous leishmaniasis caused by *L. major*. Vectors were trapped in central Tunisia during the transmission season using CDC light traps (John W. Hock Co., Gainesville, FL). A global positioning system was used to record the geographical coordinates of vector occurrence points and households tested positive for cutaneous leishmaniasis caused by *L. major*. Nine environmental layers were used as predictor variables to model the *P. papatasi* geographical distribution and five variables were used to model the *L. major* potential distribution. Ecological niche modeling was used to relate known species' occurrence points to values of environmental factors for these same points to predict the presence of the species in unsampled regions based on the value of the predictor variables. Rainfall and temperature contributed the most as predictors for sand flies and human case distributions. Ecological niche modeling anticipated the current distribution of *P. papatasi* with the highest suitability for species occurrence in the central and southeastern part of Tunisian. Furthermore, our study demonstrated that governorates of Gafsa, Sidi Bouzid, and Kairouan are at highest epidemic risk.

## Introduction

*Leishmania* parasites are the causative agents of the leishmaniases, a group of protozoan diseases transmitted to mammals, including humans, by female phlebotomine sand flies.

The disease is endemic in 98 countries with an estimated global prevalence of 12 million cases. The annual incidence of visceral leishmaniasis is estimated to be between 0.2 and 0.4 million cases, whereas cutaneous leishmaniasis affects between 0.7 and 1.2 million people each year.^1^ Leishmaniases constitute a worldwide health problem with new emerging foci due to climate and ecological changes, which are affecting the geographic distribution of leishmaniasis vectors.^2^ In arid and semi-arid areas of the Mediterranean basin, zoonotic cutaneous leishmaniasis (ZCL) is caused by the parasitic protozoan *Leishmania major* and is mainly transmitted by the predominant sand fly vector, *Phlebotomus papatasi*.^3^ The latter was formally identified as the main vector of ZCL in Tunisia,^4^ while rodents *Psammomys obesus* and *Meriones* spp. serve as the potential reservoir hosts.^5^

In Tunisia, cutaneous leishmaniasis is still a serious health problem with thousands of cases reported every year.^6^ Since its first emergence as an epidemic in Kairouan in 1982,^4^ the disease has spread in several parts of the country, particularly in the central and southern parts where 15 of 24 governorates were considered as endemic in 2006.^7^

Control of cutaneous leishmaniasis is mainly based on surveillance of incident cases and treatment. The primary prevention and prediction of the occurrence of epidemics remains a challenge because transmission is zoonotic and involves multiple factors while the tools used so far are descriptive and focus on a very limited time dimension.^5^,^7^ Studies carried out to elucidate the spatiotemporal dynamics of the disease are based on time series analysis of the incidence.^5^ However, transmission of cutaneous leishmaniasis and its spread is influenced by environmental factors affecting the reservoir and vector geographic distributions such as climate and land use.^8^ The relative importance of these factors has not been rigorously evaluated in Tunisia. During the last decade, many studies used ecological niche modeling to analyze and predict spatial patterns and distributions of vector-borne diseases such as malaria, West Nile virus infection, encephalitis, Lyme disease, lymphatic filariasis, and leishmaniasis.^9^–^11^ However, limited studies have been undertaken to assess the distribution of leishmaniasis vectors in North Africa^9^,^12^ using an ecological niche modeling approach.

The goals of this study were 1) to predict the geographic distribution of *P. papatasi* and cutaneous leishmaniasis caused by *L. major* using ecological niche modeling, 2) to assess the relative importance of environmental factors influencing the spatial distribution of *P. papatasi* and *L. major*, and 3) to estimate the population at risk of cutaneous leishmaniasis infection caused by *L. major*.

## Materials and Methods

### Study area.

Tunisia is located in the extreme north of the African continent and covers 163,610 km^2^ (N 37°20′59″-30°14′26″, E 7°31′29″-11°35′ 4″). It borders Algeria to the west, Libya to the south, and the Mediterranean Sea on the north and east sides. It includes a contrasted relief with mountainous regions in the north where the Atlas range continues from Algeria, coastal plains along Tunisia's eastern Mediterranean coast, and the desert in the southern region.

The elevation ranges from sea level, in the coastal plain, to 1,544 m, on the highest peak of the Chambi Mountain on the Tunisian dorsal. The climate varies from sub-humid in the northeastern region to a desert climate in the south of the country; annual precipitation ranges from 1,500 mm to less than 200 mm, respectively. The major difference between the northern region and the rest of the country is due to the Tunisian dorsal mountains, which separate the region with a Mediterranean climate from the arid region influenced by the desert.

The administrative boundary of Tunisia was divided into 417,690 cells of 1 × 1-km resolution as described below in section Predictor variables. The 1-km^2^ cell size was chosen because it approximated the sand flies' maximum flight distance, which is estimated to be 1 km.^13^

### Presence data.

This study used both *P. papatasi* and *L. major* occurrences obtained from intensive sampling across Tunisia. Vector presence data were obtained from surveys conducted by the Pasteur Institute of Tunis. Sand fly data were collected from 112 sampling sites representing eight governorates (Beja, Gafsa, Kairouan, Kebili, Mahdia, Sfax, Sidi Bouzid, and Sousse) across Tunisia using CDC light traps (John W. Hock Co., Gainesville, FL). The sand flies were collected for three consecutive nights per week during cutaneous leishmaniasis transmission season between May and September 2012. Identification of sand fly species was based on morphological criteria using Croset and Lewis keys.^14^,^15^ During the same surveys, confirmed human cases were obtained from health facilities, which included addresses of households with parasitologically confirmed cases of localized cutaneous leishmaniasis caused by *L. major*. Geographical positions of vector locations (*P. papatasi*) based on the trap position and the household addresses of the confirmed parasitological patients were recorded using a global positioning system. A total of 86 and 210 locations of *P. papatasi* and cutaneous leishmaniasis occurrence cases were collected, respectively (see Supplemental Appendices 1 and 2, respectively).

The study protocol was assessed by an independent scientific review committee and approved by the ethical committee of the Pasteur Institute of Tunis, whereby the head of households provided their written informed consent to be enrolled in the study. Permission to record household geographical locations and install traps on private properties was also sought from the head of households. The study was approved by the Primary Health Care Direction of the Ministry of Health.

### Predictor variables.

Twenty-four environmental layers were used as predictor variables, which were obtained from three different sources as described in Table 1. Nineteen climatic data layers representing annual trends, seasonality, and limiting environmental factors with a spatial resolution of about 1 km^2^ were collected from the WorldClim global database.^16^ Elevation, slope, aspect, and compound topographic index were derived from the GTOPO30 global digital elevation model,^17^ with a horizontal grid spacing of 30 arc seconds (∼1 km). Global land cover coverage was retrieved from the European Space Agency Global Cover Portal^13^ with 300-m pixel resolution. All environmental layers were imported into ArcGIS 10.1 software (Redlands, CA).^18^ Various processing tools were used to 1) project the layers in the UTM Zone 32N coordinate system, 2) resample to 1-km^2^ resolution, 3) clip to an area encompassing the administrative boundaries of Tunisia, and 4) convert layers to American Standard Code for Information Interchange format as long as this is the only extension that MaxEnt (Princeton, NJ) uses as input.

To avoid highly correlated and redundant information, we performed Pearson correlation tests for each pairwise combination of the 24 environmental variables.^19^ Pairs of variables with a correlation coefficient ≥ 0.9 and/or variables with a percent contribution to the model fitting less than 1% were discarded from our model calibration. For highly correlated variables, when possible, we preferred extreme variables (i.e., minimum, maximum) over mean variables, since the biological behavior of vectors is highly affected by seasonal extremes of temperature and rainfall compared to annual averages.^20^

### Population data.

Demographic data by district from the 2004 national census were obtained from the National Institute of Statistics. Later, a density population map was computed using ArcGIS 10.1 by dividing the area of each district by the respective population number. The final map represents an estimate of the number of people by ∼1-km pixel. The population at risk estimate was assessed by counting the sum of the population (from the population map) in the area of the predicted vector presence by district using a zonal statistics function.

### Ecological niche modeling.

The Grinnellian ecological niches of *P. papatasi* and *L. major* were estimated using the maximum entropy approach implemented in the MaxEnt v3.3.3 software.^21^ Maxent is freely downloadable at http://www.cs.princeton.edu/∼schapire/maxent/. By eliminating duplicate occurrence points within the same pixel, *P. papatasi* and cutaneous leishmaniasis caused by *L. major* were reduced to 76 and 124, presence points, respectively, which were randomly partitioned into 70% training data and 30% test data.

The software was used with its default parameters with 10,000 as the maximum number of background absences, 0.00001 convergent thresholds, and 500 as the maximum numbers of iterations, as suggested by Phillips and others^22^ and a logistic output presenting a continuous presence probability ranging from 0 to 1. A probability threshold representing the 10th percentile training presence points was selected as a cutoff probability used to convert continuous probability maps into binary maps (presence/absence) as suggested by Phillips and others.^23^

To account for irregular sampling of densely sampled areas, poorly sampled areas, and unsampled regions, because of feasibility constraints particularly for entomological measurements, we created a sampling bias file that was included in the Maxent settings. The bias file consisted of weighting the whole study area based on the species records as described by Elith and others.^24^ The weights are assigned to background points depending on their distance from the different occurrence points, so that both the background data and species presences become biased in the same manner leading to more valid estimates.^23^

### Evaluation of model performance.

In this study, the area under the curve (AUC) of the receiver operating characteristic (ROC) was used as a threshold independent performance criterion. The ROC curve is a graphical plot illustrating the accuracy of a binary model by varying discrimination thresholds in which the true positive rate is plotted on the *y* axis and false positive rate is plotted on the *x* axis. Hosmer and Lemeshow^25^ ranked model classification as random or with no discrimination when the AUC = 0.5, as acceptable when the AUC ranges from 0.7 to 0.8, as excellent when the AUC is between 0.8 and 0.9, and as outstanding when the AUC > 0.9. A model with a large area under the ROC curve indicates that the model is able to accurately predict presence and absence.

To assess concurrent validity of the model outputs, maximum Cohen's kappa coefficient was used as a threshold-dependent performance criterion. It is considered as the best possible accuracy achieved when varying the probability threshold. This same probability threshold was used to convert probability maps into binary maps. Compared with the percent of correct classification, kappa is a measure of categorical agreement that describes the difference between the observed and chance agreements, which can lead to a better evaluation of the model performance.^20^

Cohen's kappa coefficient was derived from the following equation:

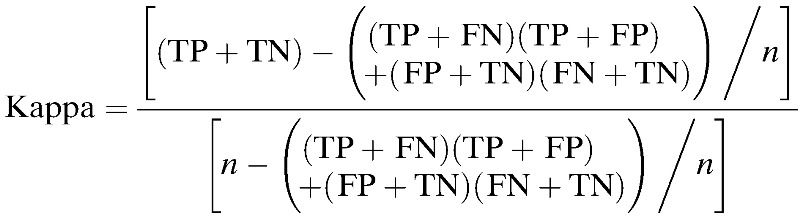
where FP is a commission error or false positive when a species is predicted as present by the model where it is absent, FN the omission error or false negative when a species is predicted as absent by the model where it is present, TP the true positive when a species is predicted as present by the model where it is present, TN the true negative when the species is predicted absent by the model where it is absent, and *n* the total number of observations used for validation.

Model performance is considered as poor when the kappa value is < 0.40, good when between 0.40 and 0.75, and excellent when > 0.75.^26^

As MaxEnt is a presence-only model, it uses background points to evaluate the model performance. It consists of taking a random sample of pixels from the study area, known as pseudo-absence points and using them in place of absences during modeling.^27^

## Results

**Figure 1.**
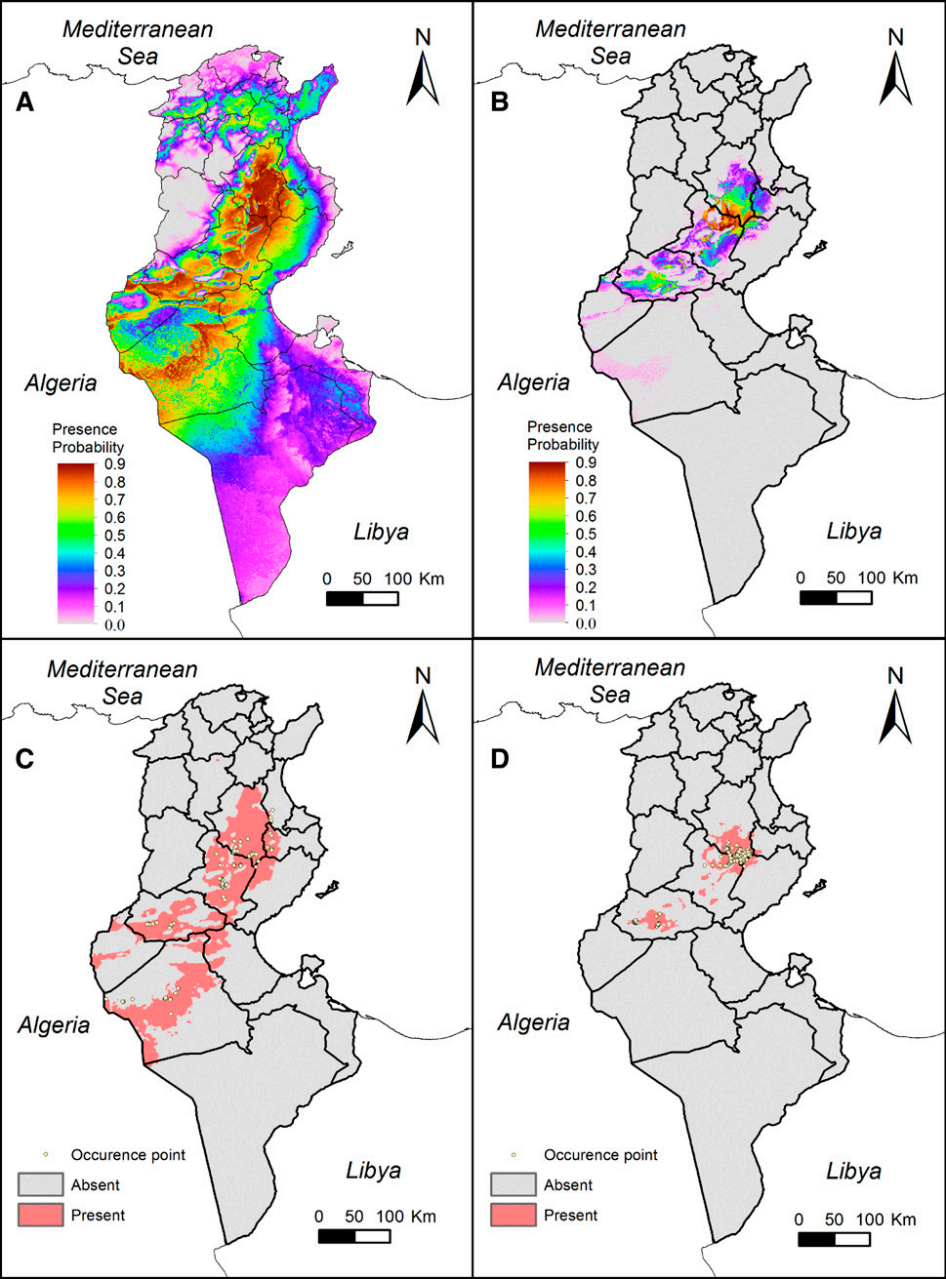
Ecological niche modeling for *Phlebotomus papatasi* and *Leishmania major* in Tunisia using the MaxEnt model. (**A**) Continuous occurrence probability map of *P. papatasi* in Tunisia. Warm colors indicate high probability of occurrence and cool colors indicate low probability of occurrence; (**B**) continuous occurrence probability map of *L. major* in Tunisia. Warm colors indicate high probability of disease occurrence and cool colors indicate low probability of disease occurrence; (**C**) binary presence/absence map of *P. papatasi* in Tunisia. Values of *P. papatasi* presence probability below the cutoff threshold (0.235) were classified as absent and values of *P. papatasi* presence probability above the cutoff threshold (0.235) were classified as present. Yellow points indicate occurrence points of *P. papatasi* used to run the model. (**D**) Binary presence/absence map of *L. major* in Tunisia. Values of *L. major* presence probability below the cutoff threshold (0.217) were classified as absent and values of *L. major* presence probability above the cutoff threshold (0.217) were classified as present. Yellow points indicate occurrence points of cutaneous leishmaniasis cases caused by *L. major* used to run the model.

Suitable habitat for *P. papatasi* and the geographical distribution of cutaneous leishmaniasis cases caused by *L. major* were mapped based on Maxent ecological niche modeling (Figure 1 A

–D). For *P. papatasi*, nine variables describing climatic (maximum temperature of warmest month BIO5, minimum temperature of coldest month BIO6, mean temperature of wettest quarter BIO8, mean temperature of warmest quarter BIO10, precipitation seasonality BIO15, precipitation of wettest quarter BIO16, and precipitation of driest quarter BIO17) and topographic (slope and elevation) variability in the study area were retained in the final model. By contrast, BIO8, BIO17, BIO5, BIO15, BIO16, and elevation were significantly associated with the presence of human cases of *L. major* (Table 2).

Table 2 shows that for the two models, when we discard one variable from the model, the AUC remains almost the same around 85% for the vector and 99% for human cases, which shows a good model performance. This does not mean that all variables contribute equally to the model performance. Indeed, as revealed in the last column (AUC with only the variable) of Table 2, the different variables contribute differently to the AUC. For example, the AUC in the model for *P. papatasi* ranged from 0.54 to 0.76 when we used the slope or the precipitation seasonality alone as predictor variables, respectively. Similarly, the same finding was noticed when modeling cutaneous leishmaniasis caused by *L. major* with an AUC varying from 0.74, when the aspect was used alone as a predictor, to 0.98, when the distribution probability of *P. papatasi* was used alone in the model.

Although topographic variables affect vector and human disease distributions, the contribution of elevation and slope to the model fitting was 19.2% for *P. papatasi*. Nevertheless, only 10.1% of the contribution was associated with elevation in the *L. major* model.

On the other hand, for *P. papatasi*, the main explanatory parameters were the precipitation of the driest quarter (26.3%), the mean temperature of the wettest quarter (20.7%), the maximum temperature of the warmest month (14%), and topographic variables (19.2%) with an overall explanation of the variance of 80.2%. For *L. major* human cases, the most important variables were mean temperature of wettest quarter (48.2%), precipitation of driest quarter (18.6%), maximum temperature of warmest month (15.6%), and elevation (10.1%) with an overall explanation of the variance of 92.5%.

AUC values were greater than 0.9 indicating that the models performed better than random and have good robustness. Kappa values for the training data are greater than 0.75, which is a good ratio for the true classification that is not affected by chance agreement. Kappa values for the test data are slightly lower than values for the training data, which can be explained by the difference in the sample size for each dataset (70% training, 30% test). This result is not surprising given that data used for training the model are expected to provide better performance.

Figure 1A and B show the potential geographic distribution of human cases of *P. papatasi* and *L. major*, respectively. For the *P. papatasi* ecological niche model, 74 presence points were located within the area suitable for the *P. papatasi* geographical distribution, and only 2 points fell outside the predicted presence areas (2.6%). Furthermore, only three presence cases were predicted as absences and fell outside the region forecasted as suitable for human cases of *L. major*, which represents only 1.4% of the whole sample.

**Figure 2.**
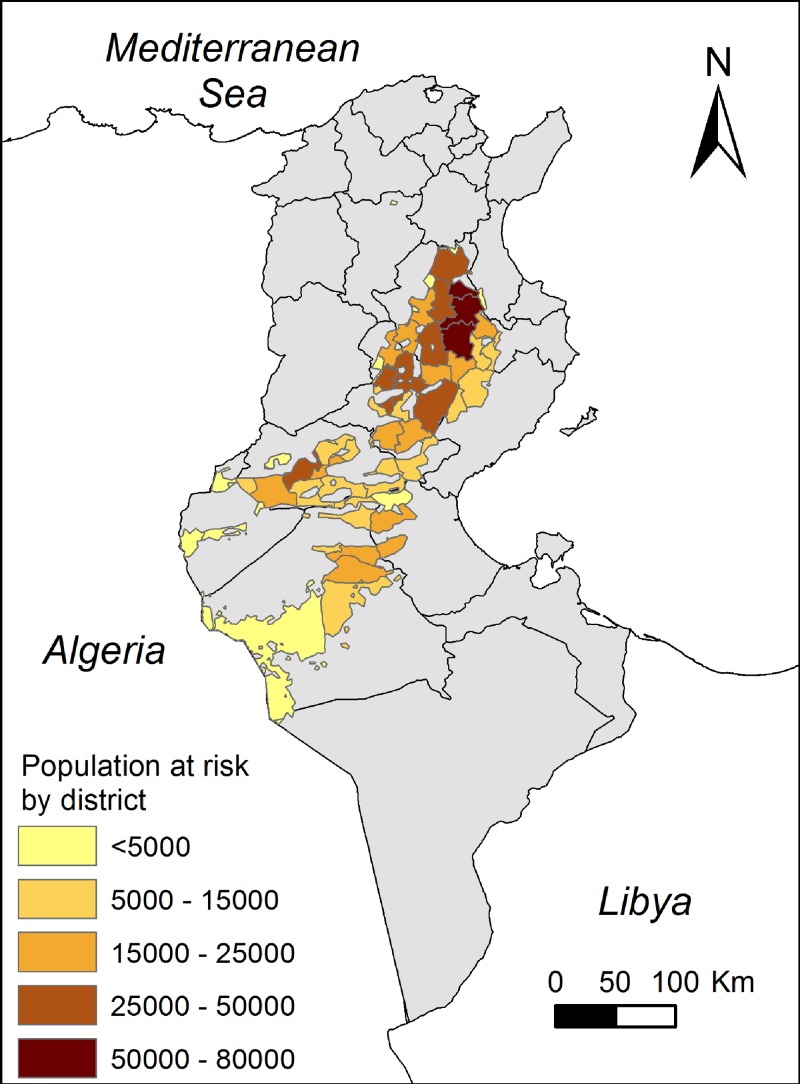
Population at risk of cutaneous leishmaniasis caused by *Leishmania major* by district.

The population at risk map is presented in Figure 2

. It represents 26% of the total population for the governorates affected by the disease in Tunisia and where MaxEnt predicted *P. papatasi* as present (846,938 inhabitants). The population estimated to be at risk in the governorates of Kairouan, Sidi Bouzid, and Gafsa, the most endemic for ZCL, represents 87% of the total population at risk.

## Discussion

In agreement with previous studies using ecological niche modeling,^9^,^10^,^12^,^28^,^29^ this study accurately predicted the spatial distribution of cutaneous leishmaniasis. To our knowledge, this is the first time that the MaxEnt model was used for this purpose in Tunisia. This tool was successfully developed and adapted to the context of this disease showing strong results. Indeed, it confirmed the geographical distribution of *P. papatasi* in the governorates of Kairouan, Sidi Bouzid, Gafsa, Kébili, and Tozeur. Surprisingly, the model predicted that the northwestern side of Sfax, the western part of Mahdia, the southern part of Zaghouan, and the western side of Gabès are at high risk for the emergence of cutaneous leishmaniasis in Tunisia. All these geographical areas neighbor the classic focus of cutaneous leishmaniasis and constitute potential extension zones of the transmission cycle. Indeed, movements of host reservoirs are responsible for the spread of the transmission cycle as demonstrated by Ghawar et al.^30^ using rodent telemetry. MaxEnt ecological niche modeling of the cutaneous leishmaniasis vector in Tunisia corroborates other findings in the same region.^31^–^33^ Moreover, the whole area predicted as suitable for disease occurrence is included in the area forecasted as having all the suitable conditions for *P. papatasi* presence (Figure 1C and D). This confirms previous work demonstrating that *P. papatasi* is the main zoonotic cutaneous leishmaniasis vector in Tunisia.^4^,^34^ Indeed, one of the criteria suggested by Claborn^35^ for incriminating vectors for disease transmission is that the specific sand fly geographic distributions must coincide with the distribution of human cases.

This study revealed that most of the variability is explained by temperature and precipitation; the role of altitude appeared to be important because of its indirect effect on temperature. In the following sections, the importance of each factor is discussed.

### Temperature.

Ambient temperature is one of the most important factors affecting developmental times and survival of sand flies.^36^ Guzmán and Tesh^37^ examined *P. papatasi* endurance under different laboratory temperatures. They concluded that the survival of adult sand flies was not detected at temperatures below 15°C. An earlier study conducted by Theodor^38^ reported that adult *P. papatasi* developed cold paralysis at 10°C and that all insects maintained at this temperature died within 19 days.

This study showed that the mean temperature of the wettest quarter significantly contributes to *P. papatasi* model fitting with 20.7%. The mean temperature of the wettest quarter varied from 3.9°C to 22.2°C over the whole study area; it only ranged from 9.4°C to 22.1°C in the area suspected as being suitable habitat for *P. papatasi*. However, this confirms the previous author's conclusions suggesting that low temperatures negatively affect *P. papatasi* longevity. There is a relatively large difference between the value of minimal temperatures for *P. papatasi* resilience suggested by Guzmán and Tesh^37^ (15°C) compared with our value (8.2°C). This discrepancy might be explained by the differences between field and laboratory conditions or sand fly species. Indeed, contrary to laboratory conditions, temperatures in the field cannot be maintained constant for a long period. Moreover, sand flies spend most of their lives in protected refuges, such as caves, wells, animal burrows, cracks in the soil, domestic animal shelters, cracked walls, and leaf litter,^39^,^40^ which protects them from long exposure to climatic extremes such as low temperatures.

Similar results were found by González and others^11^ using the MaxEnt model to predict the distribution of two leishmaniasis vectors in North America. They concluded that mean temperatures of the wettest quarter and the minimum temperature of the coldest month are the variables that contributed the most to the model fitting of *Lutzomyia anthophora* and *Lutzomyia diabolica*, respectively.

### Precipitation.

The effect of rainfall on sand fly abundance and leishmaniasis incidence has been widely studied.^7^,^41^–^45^ Nevertheless, the findings of various studies differ widely. Indeed, Gálvez and others^44^ concluded that higher densities of leishmaniasis vectors are associated with lower annual mean precipitation in Spain. According to a previous study conducted by Elnaiem and others,^42^ annual rainfall appears to be the most important predictive variable positively affecting the probability of the presence of leishmaniasis among the vector and the host in Sudan.

Several authors suggest that higher precipitation enhances the growth of plants, which provides more food and burrows for reservoir rodents, thus offering ideal sand fly habitats.^9^,^46^ Our study demonstrated that *P. papatasi* occurs only in areas where precipitation during the driest quarter is below 37 mm. In fact, intense summer rainfall can cause the flooding of rodent burrows affecting most *P. papatasi* breeding sites in Tunisia. It seems that most of the annual precipitation occurs during the wet winter season in the Mediterranean basin, which positively affects leishmaniasis vector abundance. On the other hand, extreme dry season precipitation negatively affects vector presence. This finding corroborates the results of Toumi and others,^7^ who found a negative association between rainfall above 37.34 mm and zoonotic cutaneous leishmaniasis incidence in the same study area.

### Altitude.

According to ecological niche modeling in this study, *P. papatasi* and leishmaniasis cases occurred exclusively in locations at low altitudes (< 520 m). This finding is in agreement with previous findings.^42^,^47^,^48^ In addition, other studies on the ecology of sand flies showed the importance of altitude on the distribution of sand fly species^9^,^49^–^52^; however, it seems that elevation does not directly affect the geographical distribution of *P. papatasi*. In fact, altitude is closely related to abiotic factors (such as temperature, moisture, and rainfall) and biotic factors (such as the distribution of the main sand fly host *Psammomys obesus* and vegetation). At higher altitudes, the temperature is lower (according to the thermal gradient at a rate of −0.6°C/100 m), precipitation increases and vegetation changes.^53^

### Population at risk.

The population at risk seems to corroborate records of cutaneous leishmaniasis cases from the National Control Program for Leishmaniasis as shown by statistics from the Tunisian health ministry^6^ (see Table 3). Indeed, the number of cutaneous leishmaniasis cases from 1998 to 2007 in the governorates of Sidi Bouzid, Kairouan, and Gafsa represents the majority of cases recorded in the country (77%). Nevertheless, our study estimates that Kairouan is the governorate with the highest population at risk, while the ministry statistics show that Sidi Bouzid has the highest population infected by cutaneous leishmaniasis (18,508 cases between 1998 and 2007). This discrepancy can be explained by the fact that the reporting bias is the lowest in Sidi Bouzid where awareness is very high for ZCL and a long tradition of research and intervention has been in place since 1990. Our estimate is based on the vector distribution and climatic variables, while the disease occurrence in humans is highly related to others factors, such as the presence of parasite reservoirs and socioeconomic aspects, and, particularly, the past history of disease in the community (herd immunity), which play a major role in shaping the geographic boundaries of the incidence of the human disease.

For the same reasons, we predicted that 21,183 inhabitants in the governorate of Gabès, mainly in the district of Menzel El Habib and Al-Hamma, are at risk; no leishmaniasis cases were reported until 2007 in these districts and cases have started to emerge recently. Areas predicted to be suitable for vector dispersal where no cutaneous leishmaniasis cases were reported need special attention from health authorities because human disease is particularly severe in emerging foci among naive populations.

This study was based on valid information for the confirmation of presence data for *P. papatasi* and human disease of cutaneous leishmaniasis caused by *L. major* from field observations and a large sample size. It confirmed the importance of environmental and climate factors on the distribution of leishmaniasis and demonstrated the utility of niche modeling for the prediction of the geographic spread of leishmaniasis.

Although our model performed well, several authors have criticized the use of the AUC to assess model performance,^54^,^55^ since it overestimates model performance when no absence data are used. For this reason, in future research, we recommend using the partial AUC to assess model performance, as described by Peterson and others.^56^

Despite some limitations of the tool to predict the presence of the vector in some areas particularly in the south, ecological niche modeling should be considered in the future as a valuable tool in addition to experimental laboratory studies for a better understanding of the biology of vector species. It can also be very useful for studies predicting emerging foci and exploring the impact of climate change scenarios on the dynamics of vectors and diseases.

## Supplementary Material

Supplemental Appendices.

## Figures and Tables

**Table 1:** Description and sources of environmental variables collected for the model

Environmental variables	Abbreviation	Unit	Source
Annual mean temperature	BIO1	°C	WorldClim
Mean diurnal range (mean of monthly (max temperature − min temperature))	BIO2	°C	WorldClim
Isothermality (BIO2/BIO7) (×100)	BIO3	–	WorldClim
Temperature seasonality (standard deviation × 100)	BIO4	°C	WorldClim
Max temperature of warmest month	BIO5	°C	WorldClim
Min temperature of coldest month	BIO6	°C	WorldClim
Temperature annual range (BIO5–BIO6)	BIO7	°C	WorldClim
Mean temperature of wettest quarter	BIO8	°C	WorldClim
Mean temperature of driest quarter	BIO9	°C	WorldClim
Mean temperature of warmest quarter	BIO10	°C	WorldClim
Mean temperature of coldest quarter	BIO11	°C	WorldClim
Annual precipitation	BIO12	mm	WorldClim
Precipitation of wettest month	BIO13	mm	WorldClim
Precipitation of driest month	BIO14	mm	WorldClim
Precipitation seasonality (coefficient of variation)	BIO15	mm	WorldClim
Precipitation of wettest quarter	BIO16	mm	WorldClim
Precipitation of driest quarter	BIO17	mm	WorldClim
Precipitation of warmest quarter	BIO18	mm	WorldClim
Precipitation of coldest quarter	BIO19	mm	WorldClim
Elevation	Elevation	m	Derived from GTOPO30
Slope	Slope	%	Derived from GTOPO30
Aspect	Aspect	°	Derived from GTOPO30
Compound topographic index	CTI	–	Derived from GTOPO30
Land cover	Land cover	–	European Space Agency

Max = maximum; min = minimum.

**Table 2:** Predictor variables retained for modeling the geographical distribution of *Phlebotomus papatasi* and cutaneous leishmaniasis caused by *Leishmania major*

	Variable	Percent contribution	AUC without the variable	AUC with only the variable
*P. papatasi*	BIO17	26.30	0.84	0.75
	BIO8	20.70	0.85	0.62
	BIO5	14.00	0.85	0.67
	Elevation	10.90	0.85	0.63
	Slope	8.30	0.86	0.54
	BIO10	5.20	0.85	0.78
	BIO6	5.10	0.85	0.67
	BIO15	5.00	0.86	0.76
	BIO16	4.6	0.85	0.69
Cutaneous leishmaniasis caused by *L. major*	BIO8	48.2	0.99	0.96
	BIO17	18.6	0.99	0.94
	BIO5	15.6	0.99	0.88
	Elevation	10.1	0.99	0.81
	BIO15	7.5	0.99	0.93
	Slope	5.6	0.99	0.74
	BIO16	0.5	0.99	0.92

AUC = area under the curve. Predictor variables retained for modeling the geographical distribution of *P. papatasi* and cutaneous leishmaniasis cases caused by *L. major* with the percentage of the contribution in the final model, sample average, and the AUC or receiver operating characteristic with and without the variable considering the remaining variables.

**Table 3:** Population at risk for cutaneous leishmaniasis

Governorate	Population at risk	Population	Cutaneous leishmaniasis cases 1998–2007
Kairouan	353,599	542,609	10,443
Sidi Bouzid	234,010	395,506	18,508
Gafsa	146,878	323,709	15,249
Kebili	49,860	143,218	3,617
Mahdia	44,805	377,853	2,306
Sfax	22,829	855,256	3,800
Gabès	21,183	342,630	–
Tozeur	8,617	97,526	3,014
Zaghouan	936	160,963	12
Sousse	1,611	544,413	642
Total	884,328	3,783,683	57,591

Total population according to the Tunisian National Census 2010 and cutaneous leishmaniasis cases reported to health authorities between 1998 and 2007 by governorate.
