# Supplementary material for: Ecological Niche Modeling for the Prediction of the Geographic Distribution of Cutaneous Leishmaniasis in Tunisia
Source: Am J Trop Med Hyg. 2016 Apr 6;94(4):844–51. doi: 10.4269/ajtmh.15-0345 (PMC4824228; doi:10.4269/ajtmh.15-0345)
Supplement: Supplementary file 1 [file SD5.pdf]

Supplemental Appendix 1

Phlebotomus Papatasi Occurrence Points Sampling Location

Station	Latitude	Longitude	District
1	35.386050	9.936200	Bou Hajla
2	35.209883	9.988217	Bou Hajla
3	35.279817	10.238217	Bou Hajla
4	35.386050	9.936250	Bou Hajla
5	35.255117	10.308050	Chorbane
6	35.240800	10.305517	Chorbane
7	33.489450	8.867800	Douz
8	33.493633	8.866217	Douz
9	35.168883	9.980150	Echrarda
10	35.196850	9.996533	Echrarda
11	35.190383	10.001833	Echrarda
12	35.116433	10.080283	Echrarda
13	35.110367	10.099350	Echrarda
14	35.102700	10.098367	Echrarda
15	35.197500	10.036717	Echrarda
16	35.192633	10.067350	Echrarda
17	33.168050	8.750567	Faouar
18	33.356500	8.750050	Faouar
19	33.350883	8.747883	Faouar
20	33.361433	8.663483	Faouar
21	33.357167	8.666233	Faouar
22	33.354617	8.171467	Faouar
23	33.367350	8.684433	Faouar
24	33.371683	8.674767	Faouar
25	33.432200	8.826600	Faouar
26	33.322083	8.040267	Faouar
27	33.314933	8.032083	Faouar
28	33.318483	8.024050	Faouar
29	33.349883	7.778050	Faouar
30	33.322496	7.955246	Faouar
31	34.311900	8.475083	Gafsa Sud
32	34.325233	8.536400	Gafsa Sud
33	34.317733	8.794633	Mdhila
34	34.297167	8.762683	Mdhila
35	34.302750	8.752833	Mdhila
36	34.261500	8.731750	Mdhila
37	36.659124	9.619117	Medjez El Bab
38	34.777933	9.568117	Meknassy
39	34.760167	9.580150	Meknassy
40	34.617100	9.581333	Meknassy
41	34.653250	9.558333	Meknassy
42	35.184191	10.180836	Menzel Chaker
43	34.307017	8.419450	Métlaoui
44	34.302517	8.420600	Métlaoui
45	34.307933	8.392150	Métlaoui
46	35.233883	9.778550	Nasrallah
47	35.213133	9.713083	Nasrallah
48	35.222700	9.718200	Nasrallah
49	35.231767	9.732050	Nasrallah
50	35.275700	9.736017	Nasrallah
51	35.362217	9.729483	Nasrallah
52	35.292817	9.752583	Nasrallah
53	35.352583	9.825467	Nasrallah
54	35.450483	10.286100	Ouled Chamekh
55	35.423150	10.307100	Ouled Chamekh
56	35.169800	9.957833	Ouled Haffouz
57	35.063017	9.818350	Ouled Haffouz
58	35.028817	10.060267	Regueb
59	35.057917	9.507183	Sidi Bouzid Est
60	35.058950	9.726083	Sidi Bouzid Est
61	35.044500	9.729183	Sidi Bouzid Est
62	35.049900	9.721200	Sidi Bouzid Est
63	35.055083	9.819067	Sidi Bouzid Est
64	35.044533	9.825233	Sidi Bouzid Est
65	35.197717	9.453333	Sidi Bouzid Ouest
66	35.619767	10.294917	Sidi El Hani
67	35.618550	10.304317	Sidi El Hani
68	35.657367	10.298383	Sidi El Hani
69	35.750683	10.330833	Sidi El Hani
70	35.716971	10.346947	Sidi El Hani
71	34.791133	9.574900	Souk Jedid
72	34.790583	9.575400	Souk Jedid
73	34.822267	9.599650	Souk Jedid
74	34.805133	9.602583	Souk Jedid
75	34.786083	9.558450	Souk Jedid
76	34.797533	9.549317	Souk Jedid
77	34.786650	9.541633	Souk Jedid
78	34.802783	9.524567	Souk Jedid
79	34.803933	9.536333	Souk Jedid
80	34.820533	9.539917	Souk Jedid
81	34.877050	9.525083	Souk Jedid
82	34.878033	9.536133	Souk Jedid
83	34.875483	9.540117	Souk Jedid
84	34.881483	9.519550	Souk Jedid
85	34.886883	9.504917	Souk Jedid
86	34.886650	9.499683	Souk Jedid

(continued)

Supplemental Appendix 1

Continued

Supplemental Appendix 2

Cutaneous leishmaniasis cases caused by *Leishmania major* occurrence points sampling location

Station	Latitude	Longitude	Governorate	District
1	34.4212	8.7709	Gafsa	Gafsa Sud
2	34.3891	8.7184	Gafsa	Gafsa Sud
3	34.3887	8.7193	Gafsa	Gafsa Sud
4	34.2897	8.7527	Gafsa	Mdhila
5	34.2829	8.7497	Gafsa	Mdhila
6	34.3009	8.7560	Gafsa	Mdhila
7	34.2941	8.7581	Gafsa	Mdhila
8	34.2621	8.7260	Gafsa	Mdhila
9	34.2963	8.7621	Gafsa	Mdhila
10	34.2928	8.7537	Gafsa	Mdhila
11	34.2927	8.7569	Gafsa	Mdhila
12	34.2996	8.7598	Gafsa	Mdhila
13	34.2786	8.7460	Gafsa	Mdhila
14	34.2986	8.7603	Gafsa	Mdhila
15	34.2882	8.7537	Gafsa	Mdhila
16	34.2919	8.7578	Gafsa	Mdhila
17	34.2817	8.7461	Gafsa	Mdhila
18	34.2809	8.7478	Gafsa	Mdhila
19	34.2610	8.7309	Gafsa	Mdhila
20	34.2936	8.7619	Gafsa	Mdhila
21	34.2810	8.7475	Gafsa	Mdhila
22	34.2851	8.7455	Gafsa	Mdhila
23	34.3116	8.3887	Gafsa	Métlaoui
24	34.3398	8.4067	Gafsa	Métlaoui
25	34.3196	8.4113	Gafsa	Métlaoui
26	34.3068	8.4151	Gafsa	Métlaoui
27	34.3041	8.4127	Gafsa	Métlaoui
28	34.3075	8.4100	Gafsa	Métlaoui
29	34.3103	8.3844	Gafsa	Métlaoui
30	34.3037	8.4200	Gafsa	Métlaoui
31	34.3072	8.4152	Gafsa	Métlaoui
32	34.3199	8.3806	Gafsa	Métlaoui
33	34.3106	8.3838	Gafsa	Métlaoui
34	34.3227	8.4091	Gafsa	Métlaoui
35	34.3328	8.4032	Gafsa	Métlaoui
36	34.3223	8.4105	Gafsa	Métlaoui
37	34.3063	8.4160	Gafsa	Métlaoui
38	34.3261	8.3994	Gafsa	Métlaoui
39	34.3097	8.3958	Gafsa	Métlaoui
40	34.3369	8.3988	Gafsa	Métlaoui
41	34.3107	8.3955	Gafsa	Métlaoui
42	34.3119	8.3944	Gafsa	Métlaoui
43	34.3217	8.4127	Gafsa	Métlaoui
44	34.3095	8.3958	Gafsa	Métlaoui
45	34.3259	8.4062	Gafsa	Métlaoui
46	34.3251	8.4077	Gafsa	Métlaoui
47	34.3095	8.4198	Gafsa	Métlaoui
48	34.3209	8.3913	Gafsa	Métlaoui
49	34.3237	8.4086	Gafsa	Métlaoui
50	34.3036	8.4192	Gafsa	Métlaoui
51	34.3101	8.3983	Gafsa	Métlaoui
52	34.3148	8.4176	Gafsa	Métlaoui
53	34.3220	8.4104	Gafsa	Métlaoui
54	34.3176	8.3896	Gafsa	Métlaoui
55	34.3265	8.4062	Gafsa	Métlaoui
56	34.3126	8.4082	Gafsa	Métlaoui
57	34.3064	8.4145	Gafsa	Métlaoui
58	34.3132	8.4056	Gafsa	Métlaoui
59	34.3218	8.4117	Gafsa	Métlaoui
60	34.3120	8.4073	Gafsa	Métlaoui
61	34.3033	8.4127	Gafsa	Métlaoui
62	34.3135	8.4194	Gafsa	Métlaoui
63	34.3106	8.3995	Gafsa	Métlaoui
64	34.3299	8.3990	Gafsa	Métlaoui
65	34.3173	8.3937	Gafsa	Métlaoui
66	34.3186	8.4003	Gafsa	Métlaoui
67	34.3128	8.4140	Gafsa	Métlaoui
68	34.3109	8.4044	Gafsa	Métlaoui
69	34.3102	8.4161	Gafsa	Métlaoui

(continued)

Supplemental Appendix 2

Continued

Station	Latitude	Longitude	Governorate	District
70	34.3055	8.4194	Gafsa	Métlaoui
71	34.3223	8.4084	Gafsa	Métlaoui
72	34.3090	8.3865	Gafsa	Métlaoui
73	34.3259	8.4016	Gafsa	Métlaoui
74	34.3157	8.3825	Gafsa	Métlaoui
75	34.3203	8.4097	Gafsa	Métlaoui
76	34.3103	8.3844	Gafsa	Métlaoui
77	34.3111	8.4163	Gafsa	Métlaoui
78	34.3345	8.4029	Gafsa	Métlaoui
79	34.3127	8.4175	Gafsa	Métlaoui
80	34.3273	8.4080	Gafsa	Métlaoui
81	34.3163	8.3898	Gafsa	Métlaoui
82	34.3401	8.4061	Gafsa	Métlaoui
83	34.3377	8.3988	Gafsa	Métlaoui
84	34.3365	8.3991	Gafsa	Métlaoui
85	34.3058	8.4204	Gafsa	Métlaoui
86	34.3105	8.4178	Gafsa	Métlaoui
87	34.3109	8.3958	Gafsa	Métlaoui
88	34.3292	8.3969	Gafsa	Métlaoui
89	34.3345	8.4066	Gafsa	Métlaoui
90	34.3111	8.3851	Gafsa	Métlaoui
91	34.3120	8.3886	Gafsa	Métlaoui
92	34.3101	8.4043	Gafsa	Métlaoui
93	34.3279	8.4052	Gafsa	Métlaoui
94	34.3250	8.4097	Gafsa	Métlaoui
95	34.3102	8.4108	Gafsa	Métlaoui
96	34.3132	8.3901	Gafsa	Métlaoui
97	34.3238	8.4053	Gafsa	Métlaoui
98	34.3168	8.4078	Gafsa	Métlaoui
99	34.3261	8.3833	Gafsa	Métlaoui
100	34.3070	8.4254	Gafsa	Métlaoui
101	34.3302	8.4037	Gafsa	Métlaoui
102	34.3361	8.3937	Gafsa	Métlaoui
103	34.3191	8.4046	Gafsa	Métlaoui
104	34.3376	8.3994	Gafsa	Métlaoui
105	35.2916	9.9610	Kairouan	Bou Hajla
106	35.1787	10.1637	Kairouan	Chrarda
107	35.1508	10.1052	Kairouan	Chrarda
108	35.0864	10.0627	Kairouan	Chrarda
109	35.1579	10.0378	Kairouan	Chrarda
110	35.1876	10.1285	Kairouan	Chrarda
111	35.1955	10.1515	Kairouan	Chrarda
112	35.1623	10.0618	Kairouan	Chrarda
113	35.1676	10.1580	Kairouan	Chrarda
114	35.1945	10.0712	Kairouan	Chrarda
115	35.1769	10.1116	Kairouan	Chrarda
116	35.2114	10.0058	Kairouan	Chrarda
117	35.1809	10.0908	Kairouan	Chrarda
118	35.1695	10.0329	Kairouan	Chrarda
119	35.1302	10.0803	Kairouan	Chrarda
120	35.1349	10.0736	Kairouan	Chrarda
121	35.1576	10.0853	Kairouan	Chrarda
122	35.1859	10.1564	Kairouan	Chrarda
123	35.2287	10.1287	Kairouan	Chrarda
124	35.2025	10.1477	Kairouan	Chrarda
125	35.1370	10.0864	Kairouan	Chrarda
126	35.1696	10.0098	Kairouan	Chrarda
127	35.2423	10.0136	Kairouan	Chrarda
128	35.1684	10.0897	Kairouan	Chrarda
129	35.2380	10.0148	Kairouan	Chrarda
130	35.1800	10.0800	Kairouan	Chrarda
131	35.1690	9.9377	Kairouan	Chrarda
132	35.1215	10.0296	Kairouan	Chrarda
133	35.1938	10.1304	Kairouan	Chrarda
134	35.0656	10.0233	Kairouan	Chrarda
135	35.1002	10.0329	Kairouan	Chrarda
136	35.1156	10.0356	Kairouan	Chrarda
137	35.1594	10.0385	Kairouan	Chrarda
138	35.1723	9.9671	Kairouan	Chrarda
139	35.1569	10.1494	Kairouan	Chrarda

(continued)

Supplemental Appendix 2
Continued

Station	Latitude	Longitude	Governorate	District
140	35.2082	9.9876	Kairouan	Chrarda
141	35.1468	9.9889	Kairouan	Chrarda
142	35.1937	10.0042	Kairouan	Chrarda
143	35.1190	10.0308	Kairouan	Chrarda
144	35.1386	10.0745	Kairouan	Chrarda
145	35.1889	10.1558	Kairouan	Chrarda
146	35.2080	9.9867	Kairouan	Chrarda
147	35.0957	10.0580	Kairouan	Chrarda
148	35.1826	9.9700	Kairouan	Chrarda
149	35.1677	10.1136	Kairouan	Chrarda
150	35.1952	9.9762	Kairouan	Chrarda
151	35.1399	10.0923	Kairouan	Chrarda
152	35.1064	10.0606	Kairouan	Chrarda
153	35.1789	9.9756	Kairouan	Chrarda
154	35.1082	10.0150	Kairouan	Chrarda
155	35.2024	10.1471	Kairouan	Chrarda
156	35.2086	9.9899	Kairouan	Chrarda
157	35.1475	10.0726	Kairouan	Chrarda
158	35.1433	10.0726	Kairouan	Chrarda
159	35.1336	10.0680	Kairouan	Chrarda
160	35.1854	10.0905	Kairouan	Chrarda
161	35.1994	10.0832	Kairouan	Chrarda
162	35.1750	10.1552	Kairouan	Chrarda
163	35.2040	10.1299	Kairouan	Chrarda
164	35.1498	10.0871	Kairouan	Chrarda
165	35.1774	10.0589	Kairouan	Chrarda
166	35.1663	10.1390	Kairouan	Chrarda
167	35.1495	10.0871	Kairouan	Chrarda
168	35.2352	9.7781	Kairouan	Nasrallah
169	35.2656	9.7930	Kairouan	Nasrallah
170	35.2607	9.8096	Kairouan	Nasrallah
171	35.2368	9.7968	Kairouan	Nasrallah
172	35.2302	9.7960	Kairouan	Nasrallah
173	35.2340	9.7783	Kairouan	Nasrallah
174	35.2559	9.8897	Kairouan	Nasrallah
175	35.2284	9.8161	Kairouan	Nasrallah
176	35.2097	9.8245	Kairouan	Nasrallah
177	35.3257	9.8359	Kairouan	Nasrallah
178	35.2531	9.8133	Kairouan	Nasrallah
179	35.2484	9.8081	Kairouan	Nasrallah
180	35.1972	9.8344	Kairouan	Nasrallah
181	35.0230	10.0755	Sfax	Menzel Chaker
182	35.1055	10.1102	Sfax	Menzel Chaker
183	35.1525	10.1634	Sfax	Menzel Chaker
184	35.1432	9.7195	Sidi Bou Zid	Ouled Haffouz
185	35.0256	9.7934	Sidi Bou Zid	Ouled Haffouz
186	35.0797	9.8751	Sidi Bou Zid	Ouled Haffouz
187	35.1480	9.9414	Sidi Bou Zid	Ouled Haffouz
188	35.1241	9.8817	Sidi Bou Zid	Ouled Haffouz
189	35.0239	9.8109	Sidi Bou Zid	Ouled Haffouz
190	35.0287	9.7815	Sidi Bou Zid	Ouled Haffouz
191	35.1404	9.8561	Sidi Bou Zid	Ouled Haffouz
192	35.0597	9.8416	Sidi Bou Zid	Ouled Haffouz
193	35.1391	9.8563	Sidi Bou Zid	Ouled Haffouz
194	35.0622	9.8383	Sidi Bou Zid	Ouled Haffouz
195	35.0561	9.8433	Sidi Bou Zid	Ouled Haffouz
196	35.1222	9.8638	Sidi Bou Zid	Ouled Haffouz
197	35.1530	9.9311	Sidi Bou Zid	Ouled Haffouz
198	35.1771	9.8479	Sidi Bou Zid	Ouled Haffouz
199	35.1772	9.8478	Sidi Bou Zid	Ouled Haffouz
200	35.0880	9.9229	Sidi Bou Zid	Ouled Haffouz
201	35.0616	9.8566	Sidi Bou Zid	Ouled Haffouz
202	35.0937	9.9645	Sidi Bou Zid	Ouled Haffouz
203	35.1525	9.9539	Sidi Bou Zid	Ouled Haffouz
204	35.1660	9.9671	Sidi Bou Zid	Ouled Haffouz
205	35.1652	9.8533	Sidi Bou Zid	Ouled Haffouz
206	35.0706	9.8745	Sidi Bou Zid	Ouled Haffouz
207	35.0800	9.8715	Sidi Bou Zid	Ouled Haffouz
208	35.0337	9.6990	Sidi Bou Zid	Sidi Bouzid Est
209	35.0330	9.4614	Sidi Bou Zid	Sidi Bouzid Est
210	35.0396	9.5806	Sidi Bou Zid	Sidi Bouzid Est
